# The Role of* Sauropus androgynus* (L.) Merr. Leaf Powder in the Broiler Chickens Fed a Diet Naturally Contaminated with Aflatoxin

**DOI:** 10.1155/2018/2069073

**Published:** 2018-10-01

**Authors:** Yos Adi Prakoso, Puspitasari, Chylen Setiyo Rini, Andika Aliviameita, Siti Isrina Oktavia Salasia, Kurniasih, Ahmad Fadhli Dzil Ikram, Baristha Walalangi, Kukuh Priya Utama, Muhammad Fajar Al Huda, Neneng Ayu Su'udiyah

**Affiliations:** ^1^Faculty of Veterinary Medicine, University of Wijaya Kusuma Surabaya, East Java, Indonesia; ^2^Integrated Laboratory, Faculty of Health, University of Muhammadiyah Sidoarjo, East Java, Indonesia; ^3^Department of Clinical Pathology, Faculty of Veterinary Medicine, University of Gadjah Mada, Yogyakarta, Indonesia; ^4^Department of Pathology, Faculty of Veterinary Medicine, University of Gadjah Mada, Yogyakarta, Indonesia; ^5^Undergraduate Student, Program of Medical Laboratory Technology, Faculty of Health, University of Muhammadiyah Sidoarjo, East Java, Indonesia

## Abstract

Aflatoxin (AF) is the secondary metabolite of* Aspergillus flavus* and commonly contaminates feed during storage. AF causes lowered growth rate, stress, and increased mortality in the poultry, especially for broiler industries. The aims of this study are to determine the effects of* Sauropus androgynus* (L.) Merr. leaf powder (SAP) in the chickens fed a diet naturally contaminated with AF. A total of 108 chickens are divided into 6 group: group I fed with basal diet (AF not detectable); group II fed with basal diet (AF not detectable) + 5% SAP; group III with AF (>1 ppb <50 ppb); group IV with AF (>1 ppb <50 ppb) + 5% SAP; group V with AF (>51 ppb <100 ppb) + 5% SAP; group VI with AF (>101 ppb <150 ppb) + 5% SAP. The data of the body weight, feed intake and efficiency, the relative weight of liver, kidney, spleen, bursa of Fabricius (BF), histopathology, haematological profile, haemagglutination inhibition (HI) titer, AF residue, and immunohistochemistry are collected on days 7, 14, and 21. All the data were analysed using SPSS 16. The supplementation of 5% SAP in the chickens fed a diet naturally contaminated with AF showed the potential effects of the body weight performance, haematological profile protection, increase in the cellular and humoral immune responses, reduction of AF residue in the organ, protection of liver, kidney, spleen, and BF histopathology, and increase in the immune-expression of CD4+/CD8+ lymphocytes ratio (P < 0.05). It shows that 5% SAP can be used as the alternative herbal supplementation to depress the impacts of aflatoxicosis in the broiler chickens.

## 1. Introduction

Aflatoxin (AF) is the secondary metabolite of* Aspergillus flavus* [1]. This mycotoxin is commonly found in the chicken feed. The AF contamination level in the feed usually depends on the feed storage itself [2]. The factors influence the AF level in the feed such as room temperature, humidity, storage, and transportation [3]. AF contamination leads poultry aflatoxicosis and becomes a serious problem [4].

The previous study showed that poultry aflatoxicosis causes lowered body weight, reduced feed intake and efficiency [5], increased risk of infection [6], stress, and mortality [7]. Aflatoxicosis causes the changing of haematological and serum biochemical profile [8], immunological depression, degeneration and necrosis of the liver, kidney, and lymphoid organs [9]. Mostly, those AF impacts in the previous studies were obtained by the artificial contamination using high doses. However, the reality of AF contamination in the poultry industries occurs naturally, not limited to one type of AF but also with varying doses of contamination, maybe less or higher. Therefore, it is necessary to evaluate the impacts of natural contamination of AF in the poultry industries and how to handle it.

The most important is AF found as the residue in the chicken's products that fed a diet contaminated with AF. As the residue, AF is resistant to food processing and possing risk for the human health [10]. It is necessary to develop the new method for decreasing the exposure risk of AF to face this public health problem. The traditional medicine is known to be efficient.* Sauropus androgynus* is the one of potential herbal medicine. The previous phytochemical study showed that* S. androgynus *leaf contains tannin, sterol, phenol, glycoside, flavonoid, catechol, and alkaloid [11]. Those components are used as the antidiabetic [12], a wound healing promoter [13], the anti-inflammatory agent [14], and the antioxidant [15]. The antioxidant such as the flavonoid and carotenoid inside the dried leaf of* S. androgynus *has a role as antioxidative stress and anticarcinogen, inhibiting the mutagenic genes and enzymes [16]. Both flavonoid and carotenoid are responsible for detoxification and it may be used as the immunomodulator and hepatoprotectant in the poultry aflatoxicosis.

The limitation of the previous study is not to evaluate the role of* S. androgynus* on the poultry aflatoxicosis. Therefore, the purposes of this study are to evaluate the role of* S. androgynus* in the broilers chickens fed a diet naturally contaminated with AF on the several parameters such as gross and histopathological changes, haematological profile, haemagglutination inhibition (HI) titer, AF residue, and immunohistochemistry.

## 2. Material and Methods

### 2.1. Preparation of* Sauropus androgynus *(L.) Merr. Leaf Powder (SAP)

All the experimental procedures are conducted in the Integrated Laboratory, Faculty of Health, the University of Muhammadiyah Sidoarjo from November 2017 until April 2018. The fresh leaf of* S. androgynus* is obtained from the herbal shop in Sidoarjo, East Java, Indonesia. It was washed in the tap water and dried in the room temperature. The dried leaf was powdered using an electric blender. The SAP was stored in the covered bottle at room temperature until required for use.

### 2.2. Qualitative Phytochemical Screening

The SAP measured about 0.5 gram for each qualitative phytochemical test. The SAP is screened using the standard test for several components such as tannin, phenol, saponin, alkaloid, flavonoid, glycoside, and the carotenoid. Tannin is tested by the homogenising of the SAP with distilled water and filtered, then the filtrate drops with 1% ferric chloride. The phenol is tested by the similar method in the tannin; however the ferric chloride concentration was 5%. The saponin is by the demonstration of the frothing in the filtrate after boiling. The content of flavonoid is tested by the Shinoda's, alkaloid by Mayer's, and the glycoside by Borntrager's method. However, the carotenoid content is tested by the mixing of the filtrate with chloroform and 85% sulphuric acid. The results of the qualitative phytochemical test are shown in the table (Table 1).

### 2.3. Experimental Animals

All the animal procedures in this study are approved by the ethical clearance committee from the Faculty of Veterinary Medicine, University of Gadjah Mada, Indonesia. A total of 108 one-day-old broiler chickens (DOC) strain Cobb were divided into 6 group. Chickens are treated in 24-hour light schedule (the light intensity reduced after 16 hours each day), 30°C temperature (and gradually decreased per week), and 65-70% humidity, with water and feed access ad libitum.

### 2.4. Experimental Procedure and Design

All chickens were fed with a broiler starter feed with 23% crude protein with 3200 kcal metabolizable energy. The feed composition was as described previously [17] and tested for AF free. Then, the feed was storage in the different room temperature, humidity, and period to increase the AF level naturally. The AF level in the broiler starter feed was routinely tested using* enzyme-linked immunosorbent assays *(ELISA) until obtaining several ranges of AF level (0 ppb, >1 < 50 ppb, >51 <100 ppb, and >101 <150 ppb). Based on the previous study, those AF levels of contamination were used in this study [18]. Next, the feeds with the appropriate AF level were mixed with 5% of SAP (Table 2).

In this study, a total of 108 DOC were divided into 6 group and each contains 18 chickens. Each group is placed in the colony cage and consisting of three sampling periods of 6 chickens per period. Group I was a control group and fed with basal diet (AF not detectable). Group II fed with basal diet (AF not detectable) + 5% SAP; group III with AF (>1 ppb <50 ppb); group IV with AF (>1 ppb <50 ppb) + 5% SAP; group V with AF (>51 ppb <100 ppb) + 5% SAP; group VI with AF (>101 ppb <150 ppb) + 5% SAP. The treatment was conducted for 21 days. Six chickens from each group were euthanised on days 7, 14, and 21. This research design was conducted to determine the protection limit of SAP on the AF exposure in the low, middle, and high level of contamination.

### 2.5. Vaccination

At the age of 3 days, all groups were vaccinated with Newcastle Disease (ND) using Medivac ND Hitchner B1 vaccine via ocular route according to the recommendation of the manufacturer.

### 2.6. Body Weight, Feed Intake, Feed Conversion Rate, and Relative Organ Weight Measurement

The recording of chicken's body weight and feed intake (FI) was conducted on days 7, 14, and 21. Comparison of feed efficiency was determined as feed conversion ratio (FCR) using the following formula:

(1) $$\begin{array}{c} \text{FCR}=\frac{feed\;\;intake\;\left(g\right)}{body\;\;weight\;\left(g\right)} \end{array}$$

Six chickens from each group were randomly selected, euthanised by cervical dislocation, and necropsied on days 7, 14, and 21. Only at day 21, the relative weight of the liver, kidney, and spleen, and BF was weighed and calculated using the following formula:

(2) $$\begin{array}{c} \text{Relative}\;\;\text{weight}=\frac{organ\;\;weight\;\left(g\right)}{body\;\;weight\;\left(100\;\;g\right)} \end{array}$$

After the measurement, the liver, kidney, spleen, and BF were cut and separated into 2 part. The first part was stored in the 10% neutral buffered formalin (NBF) for histopathology and immunohistochemistry and the second one in the sterile plastic and kept in the refrigerator for AF residue test using ELISA.

### 2.7. Blood and Serum Sampling

Six blood samples were collected from each group on days 7, 14, and 21 before sacrifice. The blood was collected via the right jugular vein using 1 ml syringes (26G × 1/2” (0,45 × 13 mm)). The blood for the haematological profile examination was stored in the tubes with ethylenediaminetetraacetic acid (EDTA) and kept in the refrigerator at 4°C. The blood samples were also collected inside the tubes without EDTA and kept in the room temperature until clotted and then centrifuged at 3.000 rpm for 15 minutes. The serum was stored at -20°C until tested.

### 2.8. Haematological Profile Examination

The blood samples were analysed for the haematological test such as total erythrocytes/red blood cells (RBC), total leucocytes/ white blood cells (WBC), haemoglobin (Hgb), packed cells volume (PCV), mean corpuscular volume (MCV), mean corpuscular haemoglobin (MCH), mean corpuscular haemoglobin concentration (MCHC), and differential count of leucocytes. The blood smear examination was performed by Giemsa staining and observed under the light microscope with the 1000× magnification. All the haematological tests were examined using the standard methods.

### 2.9. Haemagglutination Inhibition (HI)

The HI tests were performed on serum according to the OIE Manual of Standard Diagnostic Tests [19]. The results of the HI test were expressed as the geometric mean titer (GMT) and calculated by following formula [20]:

(3) $$\begin{array}{c} \log_{10}\text{GMT}=\frac{total\;\;\log10\;\;of\;\;sample}{number\;\;of\;\;sample} \end{array}$$

### 2.10. Enzyme-Linked Immunosorbent Assays (ELISA)

The ELISA was performed to count the total of AF level in the feed and organ of broiler chickens in this study. The samples weighed about 2 g, were extracted with methanol 70%, were homogenised in the room temperature, and were centrifuged. The supernatants were added in the well of the plates and diluted with phosphate buffer, then an aliquot (50*µ*L) was reacted with AF-peroxidase conjugate (50*µ*L) and mouse antibody solution (50*µ*L) against AF. The samples were incubated at 30 minutes at the room temperature. The plates washed with phosphate buffer, tetramethylbenzidine (50*µ*L), and urea peroxide (50*µ*L) were added and incubated at 30 minutes in the darkness. The stop reagent (100*µ*L) was added and the absorbance of the solution was measured with a 450 nm wavelength. The total of the AF level was calculated using the standard curve.

### 2.11. Histopathology and Immunohistochemistry

The organ (liver, kidney, spleen, and BF) from each group was fixed, dehydrated, and embedded in the paraffin. Thin sections (5 *µ*m) were mounted on the glass slide and stained using hematoxylin and eosin (H&E). For immunohistochemistry, thin sections of spleen and BF were mounted on the glass slide coated with polylysine. The slides were stained with monoclonal antibody for CD4+ (anti-rat CD4+, Novocastra, RTU-CD4-1F6, Cat. Number PA0427) and CD8+ (anti-rat CD8+, Novocastra, RTU-CD8-295, Cat. Number PA0183) which attend to the standard protocol of this product.

### 2.12. Morphometry

Two histopathologists analysed the histopathological slides under a blindfold condition to avoid bias. The assessment was performed using the semiquantitative scoring system from 0 to 4, as follows: (absence (0); minimal (1); mild (2); moderate (3); severe (4)). Each slide was analysed against several parameters (Table 3).

The immunohistochemical slides of spleen and BF were examined by ImageJ software against the percentages of the area that express the CD4+ and CD8+ lymphocytes. Then, the percentages area that immune-expression of CD4+ and CD8+ was measured as a ratio of CD4+/CD8+ lymphocytes.

### 2.13. Analysis Data

The data were analysed by SPSS 16 and presented as the mean ± standard of deviation (SD). The body weight, haematological profile, haemagglutination inhibition (HI) titer, and AF residue were analysed with two-way ANOVA and post hoc test; the relative organ weight was analysed with one way ANOVA and post hoc test; on the other hands, the histopathological and immunohistochemical data were analysed with the Kruskal-Wallis test and Man-Whitney U test. A probability value (*P*) of 0.05 was considered to be significant.

## 3. Results

### 3.1. Body Weight, Feed Intake (FI), and Feed Conversion Rate (FCR)

The results showed that 5% SAP has a potential effect on the body weight of broiler chickens fed a diet with naturally contaminated with AF (*P *< 0.05). The supplementation of 5% SAP showed a better increasing of body weight in groups II, IV, and V although, groups IV and V were fed a diet with AF natural contamination in (>1 ppb <50 ppb and >51 ppb <100 ppb) level. Surprisingly, group VI was not significantly different on the body weight compared with group I. However, group III shows the low performance regarding the body weight compared with the others (*P *< 0.05). The lowest level of AF contamination (>1 ppb < 50 ppb) in group III affects the body weight gain in the broiler chickens. It is supported by the lowest FI and FCR in group III compared with the others. On the other hand, the supplementation of 5% SAP in group VI with the highest AF contamination did not show the difference compared with the control regarding the FI and FCR. It proved that 5% SAP has a potential effect on the increase the body weight in the broiler chickens during the exposure of AF in (>101 ppb <150 ppb) level (Table 4).

### 3.2. Relative Organ Weight

**Figure 1:**
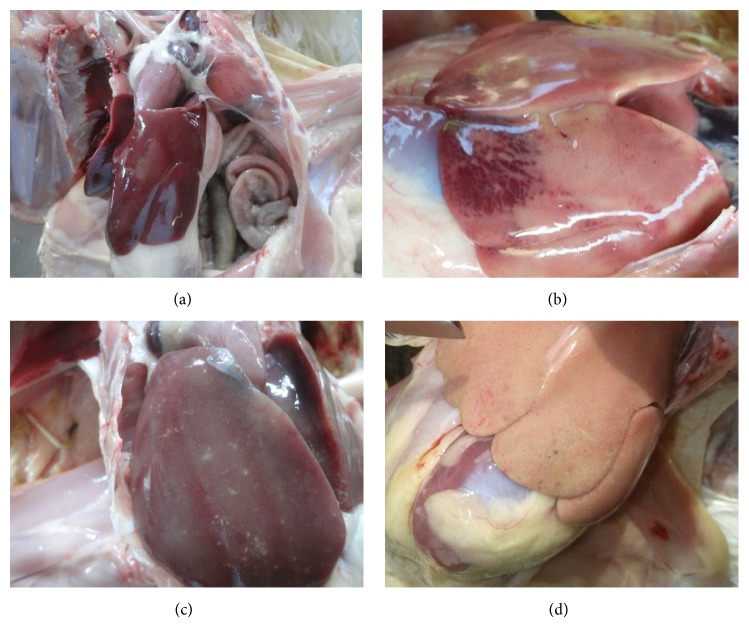
Macroscopic examination of the chicken's liver on day 21. Chicken's liver of group II showed a normal appearance with brown colour and smooth surface (6/6 samples) (a); group III showed the enlargement, obtuse angle, pale, and hemorrhage on its surface (6/6 samples) (b); group V showed the enlargement, obtuse angle, and pale (2/6 samples) (c); group VI showed a yellowish colour, and it was suspected due to fatty degeneration (1/6 samples) (d).

Macroscopically, there are no significant differences in the relative weight of liver in all groups (P > 0.05). Nevertheless, gross changing (swelling and pale) was found in the liver of group V (2/6 samples); yellowish colour in the liver of group VI (1/6 samples) and it was similar to group III (Figure 1). It was caused by the impact of high exposure of AF on the chicken's liver in groups V and VI. The high accumulation of AF in the liver changes its gross appearance, and it is confirmed by the histopathological changing that shows the fatty degeneration and bile-duct proliferation in group VI. However, it does not occur massively. The significant differences are found on the relative weight of kidney and spleen in groups III and VI (P < 0.05). The significant increasing of relative organ weight occurs on the chicken's kidney and spleen in groups III and VI. The similar result was shown on the BF; however it was marked by the significant decreasing of the relative weight of this organ in groups III and VI. However, the severe relative weight decreasing of BF was showed by group III, and it suspected due to lymphoid follicle depletion (Table 5).

### 3.3. Haematological Profile

**Figure 2:**
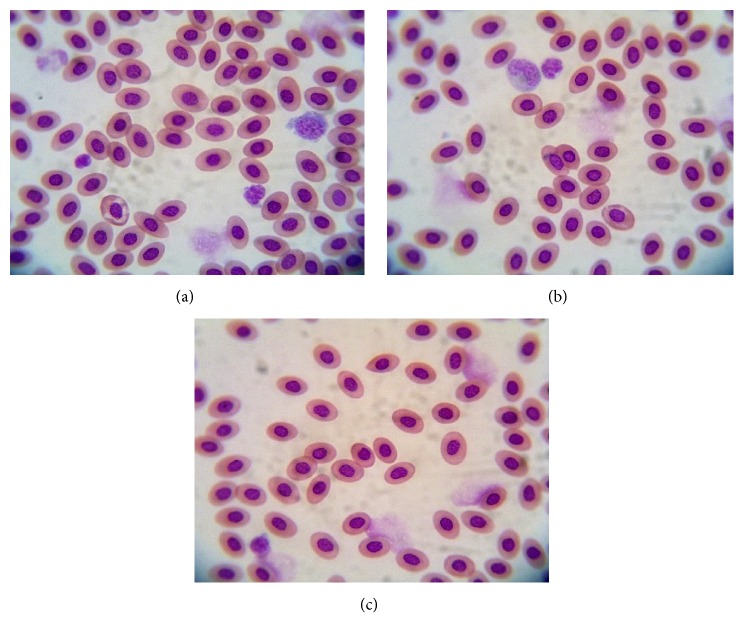
Photomicrographs of erythrocytes of the chickens fed a diet naturally contaminated with AF on day 21. The normal appearance of erythrocytes with a uniform size in group I (a); group II (b); pale colour and predominant small size of erythrocytes in group VI that indicates normocytic-hypochromic anaemia (c). Giemsa, 1000×.

This study showed that AF has potential effects on the haematological profile change in broiler chickens. Groups III and VI showed the significant differences compared with the other group regarding Hgb and MCH (*P *< 0.05). It proved that AF with low level (50 ppb and less) in the feeds has influence on the chicken's haematology and it is reciprocal with the higher level (100 ppb and more). The total RBC of group III decreases significantly (*P *< 0.05); otherwise, the total RBC between groups I and VI did not show any differences (*P *> 0.05). However, there is a significant decreasing regarding Hgb and MCH jointly in group VI indicates normocytic-hypochromic anaemia [21]. The synergic result is shown in the blood smear evaluation that indicates normocytic-hypochromic anaemia in group VI (Figure 2). The different anaemia was reported by the haematological profile in group III that indicates the macrocytic-hypochromic anaemia. It proved by the increase of the MCV value and the decreasing of the MCHC in group III (*P *< 0.05). Another significant difference on the RBC was shown by group II treated with 5% SAP without AF contamination (*P *< 0.05). Those result showed that 5% SAP could apply as a natural feed supplement for chickens. The 5% SAP supplementation can increase the total RBC in chickens. The increasing of total RBC supports the chicken's metabolism and haematology profile protection during aflatoxicosis (Table 6).

The 5% SAP supplementation on the chicken's feeds showed significant effects on the total WBC in groups II, IV, and V (*P *< 0.05). The similar effect regarding total WBC is shown in group VI and, even, did not show any significant differences compared with the control group (*P *> 0.05). There was a significant decrease regarding WBC in group III that expose to AF without SAP supplementation (*P *< 0.05). However, there are significant differences regarding bloodstream heterophils in groups IV, V, and VI, similar to group II compared with group I (*P *< 0.05). On the other hands, group III shows the significant decrease regarding circulatory heterophil compared with the others (*P *< 0.05). There are a decreasing of circulatory lymphocytes in group III (*P *< 0.05) and increasing of circulatory lymphocytes in groups II, IV, and V compared with groups I and VI (*P *< 0.05). It is proved that 5% SAP could be used to increase the cellular response in the broiler chickens with and without AF exposure. As a cellular response the ratio of H/L increased significantly in groups V and VI (*P *< 0.05); it showed that 5% SAP supports the chicken's immunology response during aflatoxicosis as protection against severe organs damage. The monocytes decrease significantly in groups II and IV (*P *< 0.05), as the response to the increase of the lymphocytes. There is the significant decrease regarding the eosinophil in groups III and VI (*P *< 0.05), the basophils in group III increase significantly compared with the others (*P *< 0.05) (Table 7).

### 3.4. Haemagglutination Inhibition

**Figure 3:**
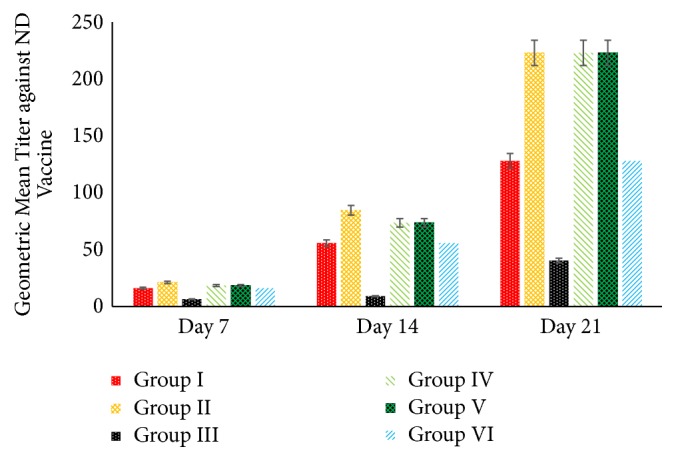
Effect of 5% SAP on the geometric mean titer (GMT) value against ND vaccine in the broiler chicken fed a diet with naturally contaminated with AF.

On day 7, the chickens in group II showed maximally increasing of the GMT against ND vaccine compared with the other groups. It is followed by groups IV and V, despite the increasing of GMT slower than group II. Surprisingly, GMT against ND vaccine in group I and group VI does not show any differences. This result proved that 5% SAP has potential effects to maintain the stability regarding titer ND production postvaccination. On the other hands, group III shows the lowest response postvaccination regarding the GMT. It is suspected caused by the impacts of AF on the broiler's lymphoid organ that impair the antibody production in group III (Figure 3).

### 3.5. Aflatoxin Residue

The AF residues in the organ (liver, kidney, spleen, and BF) in groups I, II, IV are not detected on days 7, 14, and 21. The similar results of AF residue in the organ of groups III, V, and VI were not detected at days 7, 14, and 21, except the liver at day 21. There was a detectable residue of AF in the livers of groups III, V, and VI on day 21. It may because of the accumulation effects of AF in these group. However, the detected AF level in the liver of group V was 0.10 ppb ± 0.09 (4/6 samples), and in group VI was 0.24 ppb ± 0.09 (6/6 samples). On the other hands, group III shows the highest AF residue in 0.28 ppb ± 0.58 (6/6 samples). The supplementation of 5% SAP on the chicken's feed may have resulted in the reduction of AF residue in the liver and the other organs.

### 3.6. Histopathology and Immunohistochemistry

**Figure 4:**
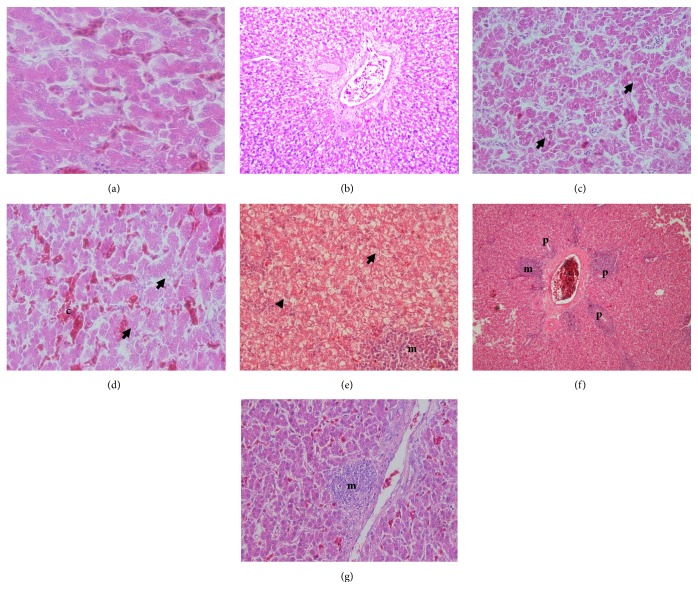
Effect of 5% SAP on the liver histopathology in the broiler chicken fed a diet with naturally contaminated with AF on day 21. The normal appearance of liver hepatocytes in group II (a); severe lipid degeneration with mononuclear cells infiltration in group III (6/6 samples) (b); lipid droplet (arrow) inside the cytoplasm of hepatocytes due to lipid degeneration in group IV (1/6 samples) (c); lipid degeneration (arrow) with sinusoid congestion (c) in group V (2/6 samples) (d); lipid degeneration (arrow) and hydrophobic degeneration (arrowhead) with mononuclear cells infiltration (m) in group V (2/6 samples) (e); bile-duct proliferation (p) and mononuclear cells infiltration (m) on the portal triad area in group VI (3/6 samples) (f); local mononuclear cells infiltration (m) in group VI (3/6 samples) (g). H&E, 10× (b, f); 40× (e); 100× (c, g); 1000× (a, d).

In this study, group III with AF (>1 ppb < 50 ppb) shows severe liver necrosis and degeneration compared with the others (*P *< 0.05). The liver necrosis and degeneration in group III started on day 7 and increased on days 14 and 21 during the AF exposure (*P *< 0.05). Based on the results, there are no the differences between group I and the others, except group III regarding liver necrosis and degeneration (*P* > 0.05). The similar results regarding perilobular and interlobular inflammation and bile-duct proliferation significantly are showed in group III compared with the others (*P *< 0.05). It proved that even the minimal doses of AF contamination take an impact on the liver histopathological change (Table 8). There are no differences on the liver histopathology (inflammation and bile-duct proliferation) on days 7 and 14 between groups I, II, IV, V, and VI (*P* > 0.05). The liver inflammation (perilobular and interlobular) and bile-duct proliferation in group V increase at day 21; however it is not as severe as group III. The 5% SAP has a potential effect on the liver protection during AF exposure (Table 8). The photomicrographs of the liver are shown in Figure 4.

**Figure 5:**
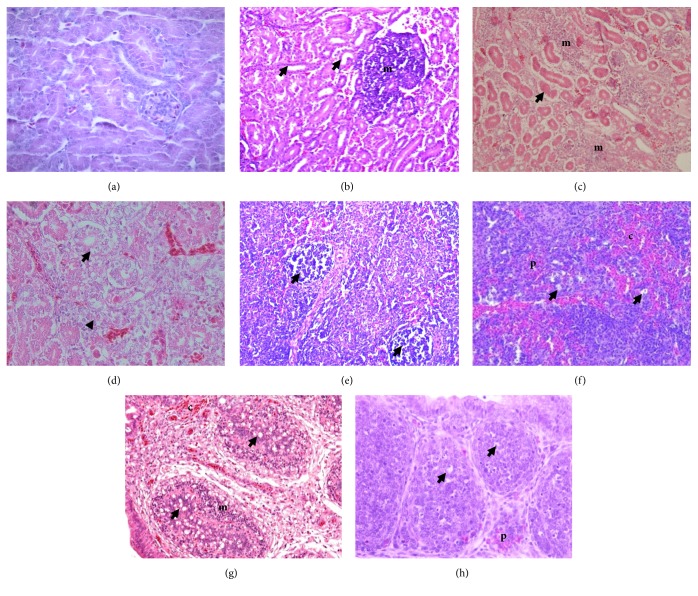
Effect of 5% SAP on the kidney, spleen, and BF histopathology in the broiler chicken fed a diet with naturally contaminated with AF on day 21. The normal appearance of kidney in group II (a); mononuclear cells infiltration (m) in the interstitial with necrosis (arrow) of the tubules epithelial of kidney in group III (b); and group V (c); necrosis (arrow) and degeneration (arrowhead) of the tubules epithelial of kidney in group VI (d); severe depletion (arrow) in the white pulp of the spleen in group III (e); massive congestion (c), depletion of lymphocytes (arrow), with polymorphonuclear cells infiltration (p) in the white pulp of the spleen in group VI (f); depletion of lymphoid follicle (arrow), extensive mononuclear cells infiltration (m), and congestion (c) of the BF in group III (g); irregular size of lymphoid follicles due to depletion of lymphocytes (arrow) with polymorphonuclear cells infiltration (p) in the BF in group VI (h). H&E, 40× (b); 100× (d, e, g); 1000× (a, c).

Group III shows the most severe kidney histopathological change (necrosis, degeneration, and inflammation) compared with the others (*P *< 0.05). The increasing of inflammatory cells infiltration is shown in groups V and VI on days 14 and 21 (*P *< 0.05). However, the necrosis and degeneration of kidney tubule in groups V and VI have not any significant difference compared with groups I, II, and IV (*P *> 0.05) (Table 9). Spleen and BF histopathological examination showed that there are no differences in the all groups (*P *> 0.05), only five (four from group III and one from group VI) from a total 108 samples of spleen in this study that showed the severe depletion of pulp and being accompanied by the congestion and inflammation; and only seven (six from group III and one from group VI) from a total 108 samples of BF that showed the depletion of lymphoid follicle, inflammation, and congestion. The spleen and BF sample that show the severe histopathological changing were from groups III and VI on day 21 (Figure 5). This results showed that 5% SAP supplementation in the broiler feed could be used to depress the impacts of AF and increase the broiler performances.

**Figure 6:**
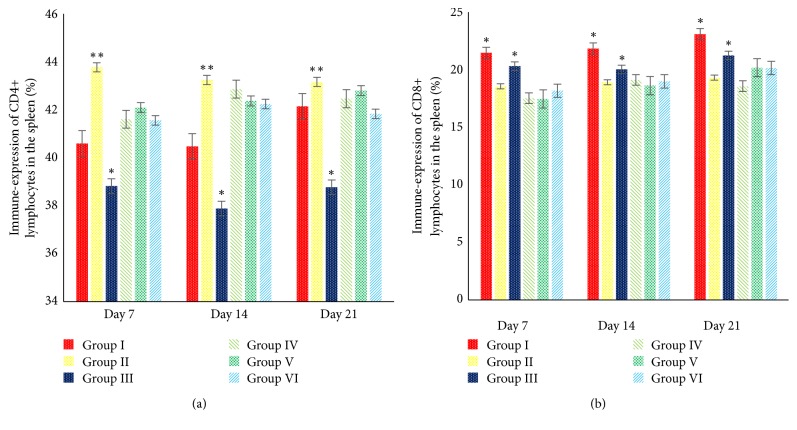
Effects of 5% SAP on the immune-expression of CD4+ (a) and CD8+ (b) lymphocytes in the spleen of broiler chickens fed a diet naturally contaminated with AF at 7, 14, and 21 days of age. *∗*/*∗∗*The different superscript showed significantly different values (*P *< 0.05).

**Figure 7:**
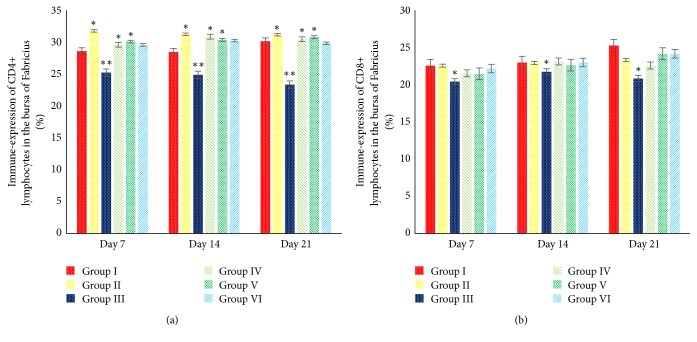
Effects of 5% SAP on the immune-expression of CD4+ (a) and CD8+ (b) lymphocytes in the BF of broiler chickens fed a diet naturally contaminated with AF at 7, 14, and 21 days of age. *∗*/*∗∗*The different superscript showed significantly different values (*P *< 0.05).

**Figure 8:**
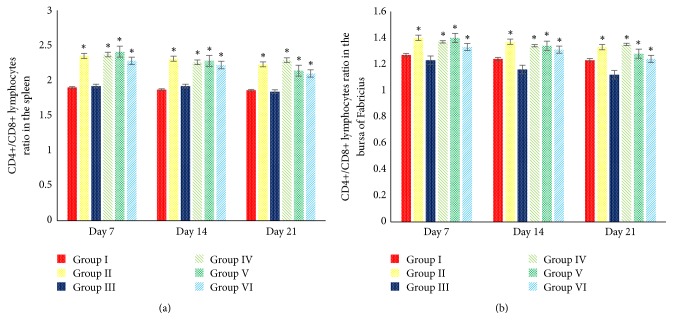
Effects of 5% SAP on the ratio of CD4+/CD8+ lymphocytes in the spleen (a) and BF (b) of broiler chickens fed a diet naturally contaminated with AF at 7, 14, and 21 days of age. *∗*The different superscript showed significantly different values (*P *< 0.05).

**Figure 9:**
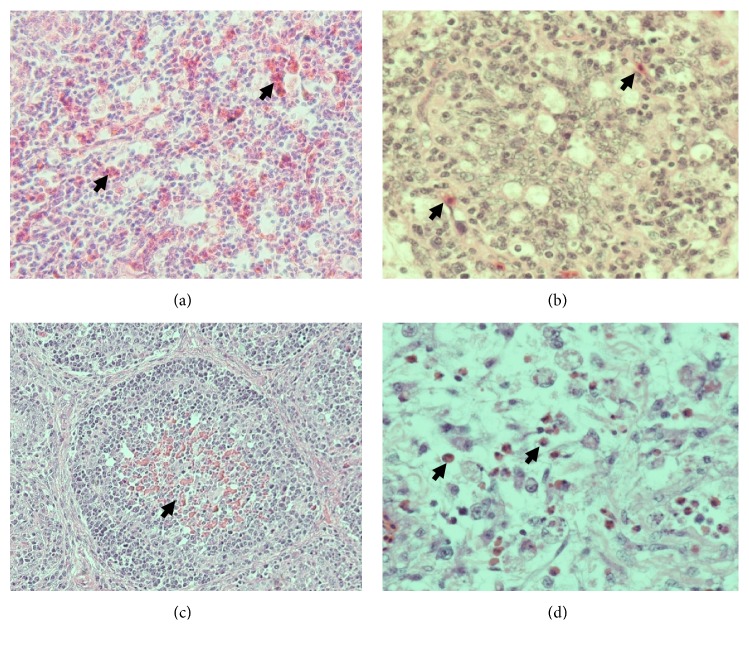
Effect of 5% SAP on the immune-expression of CD4+ and CD8+ lymphocytes in the spleen and BF of the broiler chicken fed a diet with naturally contaminated with AF on day 21. High immune-expression of CD4+ lymphocytes (arrow) (a); low immune-expression of CD8+ lymphocytes (b) in the white pulp of the chicken's spleen in group V; high immune-expression of CD4+ lymphocytes (arrow) (a); low immune-expression of CD8+ lymphocytes (b) in the lymphoid follicle of the chicken's BF in group V. IHC antibody anti-CD4+, DAB, 100× (a), 1000× (b); IHC antibody anti-CD8+, DAB, 100× (c), 1000× (d).

Spleen and BF was the prominent immunological organ. Inside the spleen and BF parenchyma the T-cells subsets can be found, such as CD4+ and CD8+ lymphocytes. The minimal doses of AF (>1 ppb <50 ppb) exposure affect the immune-expression of both CD4+ and CD8+ lymphocytes. It is proved by the results of this study that show the decrease in CD4+ and CD8+ lymphocytes in the spleen and bursa of Fabricius (*P *< 0.05). This study showed that 5% SAP supplementation in the broilers increases the immune-expression of CD4+ lymphocytes in the spleen in group II (*P *< 0.05) (Figure 6(a)) and increases the immune-expression of CD4+ lymphocytes in the BF in groups II, IV, and V (*P* < 0.05) (Figure 7(a)). However, there is no significant difference regarding the immune-expression of CD8+ lymphocytes in the BF in groups I, II, IV, V, and VI (*P *> 0.05) (Figure 7(b)). All group treated with 5% SAP has a better ratio of CD4+/CD8+ lymphocytes in the spleen and BF compared with groups I and III (*P *< 0.05) (Figures 8(a) and 8(b)). This result proves that 5% SAP supplementation has protective effects on the immunological organ in the aflatoxicosis. High immune-expression of CD4+ and CD8+ lymphocytes in the spleen and BF occurred in the centre of white pulp and lymphoid follicle (Figure 9).

## 4. Discussion

AF is a mycotoxin produced by* A. flavus* and can contaminate the wide of commodities. The majority of AF contaminates the based component of chicken's feed manufacture and has an impact on the feed intake and growth [22]; decrease egg and meat production [23]. Reducing the impacts of AF on the chickens needs the new supplementation based on the herbal formulation, such as* S. androgynus*. This study proved that 5% SAP increases the body weight of the broiler chickens in the group with and or without AF contamination. The increasing of the body weight in the broiler chickens fed a diet with 5% SAP supplementation showed better growth performance that indicates the high muscle mass, decreasing both the FI and FCR compared with the control group. It indicates the high chicken performance on the muscle mass formation. This finding was supported by the previous study that shows the biological nutritive value inside the SAP effects on the resist diseases and enhances nonspecific immune response and growth performance [24] and increases the broiler meat quality [25].

One of the AF potential effects is mutagenic, teratogenic, and hepatocarcinogenic [26]. The histopathological change during aflatoxicosis could be identified macroscopically by the relative weight examination of the liver and another internal organ such as kidney and lymphoid organ. Liver, kidney, and lymphoid organs are considered to be the target organ in poultry aflatoxicosis [27]. AF makes several macroscopical lesion such as increasing the relative weight of liver and kidney and decreases the lymphoid organ [28]. This study showed that the significant increase of the relative weight of kidney and spleen and decreasing of BF occur in groups III and VI on day 21; however, it has not in the other groups (Table 5). This study proves that SAP has potential effects on the protection of the internal organ during aflatoxicosis. It showed by the minimal macroscopical change on the liver, kidney, spleen, and BF in group VI that exposure to high level of AF contamination (>101 ppb <150 ppb) with 5% SAP supplementation.

Every nutrient produced through the metabolism process is circulated by the RBC. In the poultry aflatoxicosis, the AF depressing the haemopoietic tissues might result in decreasing of RBC production [29]. The abnormalities of RBC caused by the plasma osmolarity change. In this study, the minimum level of AF (>1 ppb <50 ppb) in group III changes the chicken's haematological profile, such as macrocytic-hypochromic anaemia. It may be caused by the increasing number of the circulatory reticulocytes on the bloodstream as a compensatory response to the tissue nutritive requirements. On the other hand, the 5% SAP supplementation has increased the total RBC significantly and maintain the total RBC in the group fed a diet naturally contaminated with AF compared with the control. The increasing of total RBC in the chickens has a potential effect on the immunity formation because avian RBC can participate in the immune response by producing cytokine-like factor [30]. RBC takes the prominent role as the transporter of oxygen to the entire body; therefore, the high number of RBC is synergic with the high oxygen in the plasma. The oxygen is just like a sandwich that attaches to erythrocytes with supported by the haemoglobin. AF (100 and 150 ppb) can decrease the Hgb concentration in the broiler, and it is mentioned by the previous study [8]. However, the 5% SAP supplementation in the broiler's feed maintains the stability of Hgb during AF exposure because of its Fe (iron) content which supports the Hgb formation [31]. The differences regarding the Hgb and MCH are decreasing in group VI due to impaired iron absorption [32]; however, the normocytic-hypochromic anaemia was a physiological defend form during the chronic exposure in the liver caused by the toxin (especially AF) as well as parasitic liver infection [33]. Activation of cytokine during the chronic infection, such toxicosis, and the reticuloendothelial systems induce changes in iron homeostasis and erythrocytes life spans contribute to the pathogenesis of normocytic-hypochromic anaemia [34, 35].

In contrast to erythrocytes, leucocytes do not exhibit lifespan in the circulatory system but rather leave the circulatory system and infiltrate to the tissues at random times in response to chemoattractant stimuli [36]. During the aflatoxicosis, there is some reduction of the total WBC in group III that is similar to the previous study [37]; however, the significant increasing of total WBC in the group with 5% SAP supplementation indicates that there is a cellular response to the agent of infection, inflammation, aflatoxicosis, and also ND vaccination in this study. It shows that SAP supports the defensive mechanism of the body from the severe injury during AF exposure and ND vaccination by activating the cellular response in the bloodstream. Heterophils are the main leucocytes that firstly increase during the pathological mechanism in the host body. Heterophils mediate opsonisation during phagocytosis [38]. Increasing the heterophils in the bloodstream is the prominent role to inflammation because heterophils granules contain antimicrobial protein, peptides, and toxic mediators [39]. However, the long-term increase in circulatory heterophils affects the ROS [40], if it increases without being offset by an increase of lymphocytes. By the increasing of lymphocytes, the host body can produce the memory and antibody to prevent the second infection from the same agent [41, 42]. In this study, the lymphocytes increase significantly to produce the antibody of ND without being affected by the AF exposure. Generally, poultry aflatoxicosis affects the decreasing of the lymphocytes and the titer antibody production after vaccination [43]. It shows that 5% SAP supplementation in feed has a prominent role in mediating lymphocytes to support the antibody production and it is proved by the stability of GMT value against ND vaccine.

The liver is the primary organ in detoxification during aflatoxicosis. In the liver, the toxin especially AF is absorbed and transformed into its metabolites form. The metabolites form of AF binds the nucleic acids and protein of the hepatocytes, causing the liver residue [44]. Long-term exposure of AF in the low levels increases the risk of the residue of AF [45]. It was similar to the result of this study that showed that the minimal AF residue was detected on the liver of broiler chickens in group III (0.28 ppb ± 0.58), group V (0.10 ppb ± 0.09), and VI (0.24 ppb ± 0.09) on day 21. The minimal residue of AF in the liver may be due to the level of AF exposure in this study and or the capability of SAP as the antioxidant, used as the detoxifying agent that reduces the bioavailability of AF. Even there was no previous study mentioning the activity of SAP as an antitoxin in poultry aflatoxicosis, however phenolic content in the SAP has a prominent role as cupric ion chelating activities and free radical detoxifying [46]. Another study showed that SAP could be used to reduce food poisoning [47].

The low levels of AF contamination in the feed (50 and 100 ppb) with chronic exposure were reported as a source of liver histopathological change [18]. However, the 5% SAP supplementation in the broiler chickens diet in this study protects those organs from the severe damage during AF exposure. In the microscopic examination, the liver necrosis and severe degeneration in all groups except group III were no found; however, the inflammation and bile-duct proliferation were observed minimally. The similar results are observed in the histopathology of kidney and lymphoid organs. It indicates that SAP has a prominent role as the antioxidant that inhibits the reactive oxygen species (ROS). ROS was the prominent mediator of cellular necrosis and degeneration [48]. ROS is impaired on the Na+ and K+ in the sodium pump and the ion exchange [49]. It causes the cellular retention and increase of the lipid droplets in the cytoplasm of the cells in several organs such as liver [50], spleen, BF, and kidney [51]. The inhibition of ROS by the antioxidant inside the SAP may increase the cellular metabolism, regeneration, and detoxification during AF exposure. Those positive effects are caused by the flavonoid and phenolic content of the SAP (Table 1). Both flavonoid and phenol are the natural antioxidants inside the SAP [52, 53].

Both CD4+ and CD8+ lymphocytes have a prominent role in the activation of the immunological complex via major histocompatibility complex (MHC) class II and class I. In aflatoxicosis, there is a changing of T-cells subpopulation in the primary lymphoid tissues (thymus and BF) [54]; however, the secondary lymphoid tissues such spleen are also affected. Similar to infectious bursal disease viruses (IBDV) infection, aflatoxicosis causes the changing of CD4+ and CD8+ lymphocytes infiltration. Commonly, both CD4+ and CD8+ lymphocytes mature in the thymus [55]; however, during AF exposure there is a dramatical change of T cells to infiltrate and repopulate the depletion B cells in the BF. Poultry aflatoxicosis reduces humoral and cellular immune response and responds poorly to vaccination. Representation regarding CD4+ and CD8+ lymphocytes is used as the indicator of cell-mediated immunity (CMI) [56]. CD4+lymphocytes were the prominent lymphocytes that produce a signal to the B cells in the BF to promote antibody production [57]. The result of this study shows that 5% SAP supplementation has a positive effect on the high GMT production against ND vaccine and it is synergic to the group with high immune-expression of CD4+ lymphocytes. On the other hand, the subpopulation of CD4+ lymphocytes in the spleen has the role as the defensive to maintain the spleen pulp integrity. Different from CD4+ lymphocytes that produce the signal, CD8+ lymphocytes are the cytotoxic cells that control the regulation and promote lysis of the destructed cells and tissues [58, 59]. The ratio of CD4+/CD8+ lymphocytes increases maximally in the group with 5% SAP supplementation as a response to protect the organ from the destructive effects of AF. The chlorophyll of the SAP is the one prominent component except for flavonoid and phenol that play a prominent mechanism to inhibit the oxidative stress [15]. Another study suggests that chlorophyll has a role as the chelating agent against oxidation and decomposition of hydroperoxides [60]. In some cases, chlorophyll has been used to induce the leucocytes production in leucopenia [61] and treat the gastrointestinal problem, anaemia, and hydroxyl radical-scavenging [62]. This study demonstrated that 5% SAP supplementation in the chicken's diet has a potential role as the immunomodulator to activate the immune-expression of CD4+ and CD8+ lymphocytes in both spleen and BF. The utilisation of 5% SAP in the feed as the supplement to increase CD4+, CD8+, and CD4+/CD8+ lymphocytes ratio as well is the relevant strategy to develop the poultry industry to face the AF problem.

## 5. Conclusion 

This study clearly demonstrated that 5%* Sauropus androgynus *leaf powder (SAP) has a potential effects of increasing the body weight performance, haematological profile protection, the cellular and humoral immune responses, reduction of AF residue in the organ, protection of liver, kidney, spleen, and BF histopathology, and increase in the immune-expression of CD4+/CD8+ lymphocytes ratio (*P *< 0.05).

## Figures and Tables

**Table 1:** Qualitative phytochemical analysis of SAP.

Variable						
Tannin	Phenol	Saponin	Alkaloid	Flavonoid	Glycoside	Carotenoid
+	+	+	+	+	+	+

+ = present; – = absent.

**Table 2:** AF level in the chicken's feed from days 0 to 21 (ppb) during the study.

Day	Group (AF level)					
	I	II	III	IV	V	VI
0	-	-	25.21	20.00	51.02	110.07
3	-	-	27	29.08	63.77	129.82
6	-	-	23	17.85	70.00	107.73
9	-	-	27.12	23.19	74.30	126.25
12	-	-	26.84	29.41	78.26	136.15
15	-	-	33.01	35.24	78.26	144.52
18	-	-	40.15	39.44	81.10	138.66
21	-	-	42.87	44.98	84.16	141.00
Mean	-	-	30.65 ± 7.30	29.89 ± 9.53	72.60 ± 10.84	129.27 ± 13.87

	Feed treatments to reach the suitable AF level					

Temp. (°C)	5	5	40	40	40	40
Humidity (%)	50	50	90	90	90	90
Incubation period (day)	-	-	7	7	14	21

SAP (%)	0	5	0	5	5	5

– = AF not detectable/without incubation.

**Table 3:** Semiquantitative scoring systems for poultry aflatoxicosis.

Organ	Parameters
Liver	Necrosis, perilobular and inflammation, hydropic and/or fatty degeneration, bile-duct proliferation
Kidney	Necrosis, inflammation, degeneration
Spleen	Depletion of pulp, inflammation, congestion
BF	Depletion of the lymphoid follicle, inflammation, congestion

**Table 4:** Effects of 5% SAP on the body weight, FI, and FCR of broiler chickens fed a diet naturally contaminated with AF at 7, 14, and 21 days of age.

Parameter	Group	Day		
		7	14	21
Body weight (g)	I	120.27 ± 2.70*∗*	338.67 ± 14.49*∗*	683.38 ± 13.32*∗*
	II	127.13 ± 3.03*∗∗*	353.40 ± 5.95*∗∗*	695.89 ± 12.45*∗∗*
	III	108.95 ± 5.51	309.83 ± 7.26	641.19 ± 22.50
	IV	125.44 ± 3.43*∗∗*	355.71 ± 4.79*∗∗*	695.80 ± 8.73*∗∗*
	V	127.13 ± 2.23*∗∗*	353.22 ± 4.97*∗∗*	690.13 ± 16.55*∗∗*
	VI	120.16 ± 2.44*∗*	337.22 ± 11.25*∗*	682.53 ± 14.11*∗*

FI (gram)	I	171.00 ± 0.89*∗*	562.33 ± 2.33*∗*	1217.33 ± 11.69*∗*
	II	171.00 ± 0.89*∗*	563.00 ± 1.67*∗*	1213.00 ± 7.69*∗*
	III	164.83 ± 4.07	539.83 ± 15.06	1155.16 ± 51.80
	IV	170.83 ± 1.47*∗*	562.33 ± 1.86*∗*	1215.33 ± 14.50*∗*
	V	169.33 ± 1.50*∗*	562.50 ± 1.51*∗*	1213.33 ± 4.30*∗*
	VI	172.00 ± 2.00*∗*	564.17 ± 3.54*∗*	1226.00 ± 6.75*∗*

FCR	I	1.42 ± 0.03*∗*	1.66 ± 0.07*∗*	1.78 ± 0.03*∗*
	II	1.34 ± 0.03*∗∗*	1.59 ± 0.02*∗∗*	1.74 ± 0.04*∗∗*
	III	1.51 ± 0.09	1.74 ± 0.03	1.80 ± 0.06
	IV	1.36 ± 0.03*∗∗*	1.58 ± 0.02*∗∗*	1.74 ± 0.02*∗∗*
	V	1.33 ± 0.02*∗∗*	1.59 ± 0.02*∗∗*	1.75 ± 0.04*∗∗*
	VI	1.43 ± 0.03*∗*	1.67 ± 0.05*∗*	1.79 ± 0.03*∗*

*∗*/*∗∗*The different superscript on the same column showed significantly different values (*P *< 0.05).

**Table 5:** Effects of 5% SAP on the relative organ weight of broiler chickens fed a diet naturally contaminated with AF at 21 days of age.

Group	Relative organ weight (g/100 g body weight)			
	Liver	Kidney	Spleen	BF
I	2.26 ± 0.06	0.64 ± 0.03	0.14 ± 0.03	0.30 ± 0.01
II	2.29 ± 0.01	0.64 ± 0.02	0.14 ± 0.02	0.31 ± 0.02
III	2.31 ± 0.02	0.71 ± 0.01*∗*	0.22 ± 0*∗*	0.18 ± 0.02*∗∗*
IV	2.25 ± 0.04	0.65 ± 0.02	0.14 ± 0.02	0.30 ± 0.01
V	2.26 ± 0.38	0.64 ± 0.03	0.15 ± 0.02	0.30 ± 0.05
VI	2.29 ± 0.05	0.70 ± 0.01*∗*	0.20 ± 0.01*∗*	0.23 ± 0.05*∗*

*∗*/*∗∗*The different superscript on the same column showed significantly different values (*P *< 0.05).

**Table 6:** Effects of 5% SAP on the haematological profile (RBC, PCV, Hgb, MCV, MCH, and MCHC) of broiler chickens fed a diet naturally contaminated with AF at 7, 14, and 21 days of age.

Parameter	Group	Day		
		7	14	21
RBC (× 10^6^/ *µ*L)	I	2.17 ± 0.08	2.40 ± 0.03	2.74 ± 0.06
	II	2.25 ± 0.04*∗*	2.50 ± 0.06*∗*	2.85 ± 0.05*∗*
	III	2.10 ± 0.17*∗∗*	2.09 ± 0.05*∗∗*	2.18 ± 0.09*∗∗*
	IV	2.23 ± 0.03	2.39 ± 0.19	2.81 ± 0.07
	V	2.13 ± 0.07	2.40 ± 0.08	2.64 ± 0.07
	VI	2.24 ± 0.05	2.40 ± 0.08	2.63 ± 0.14

PCV (%)	I	26.92 ± 0.48	27.41 ± 0.34	28.74 ± 0.41
	II	27.31 ± 0.40*∗*	28.23 ± 0.35*∗*	28.72 ± 0.32*∗*
	III	27.27 ± 0.30	27.87 ± 0.19	27.25 ± 0.34
	IV	27.26 ± 0.29*∗*	28.04 ± 0.45*∗*	28.46 ± 0.39*∗*
	V	27.07 ± 0.28*∗*	28.21 ± 0.39*∗*	28.58 ± 0.30*∗*
	VI	27.21 ± 0.33*∗*	28.08 ± 0.33*∗*	27.05 ± 0.84*∗*

Hgb (g/dl)	I	10.36 ± 0.27	10.72 ± 0.18	11.22 ± 0.09
	II	10.52 ± 0.23	10.80 ± 0.08	11.33 ± 0.07
	III	10.22 ± 0.13*∗*	10.25 ± 0.17*∗*	10.40 ± 0.30*∗*
	IV	10.52 ± 0.24	10.77 ± 0.02	11.29 ± 0.15
	V	10.52 ± 0.31	10.69 ± 0.31	11.16 ± 0.24
	VI	10.36 ± 0.34*∗*	10.40 ± 0.26*∗*	10.90 ± 0.22*∗*

MCV (fl)	I	124.16 ± 5.57	113.86 ± 1.79	104.96 ± 3.02
	II	121.44 ± 3.82	112.92 ± 3.79	100.84 ±2.75
	III	129.67 ± 9.97*∗*	133.28 ± 4.30*∗*	125.09 ± 4.82*∗*
	IV	122.20 ± 2.89	117.87 ± 11.57	101.18 ± 3.32
	V	126.98 ± 5.84	117.64 ± 4.86	108.12 ± 3.10
	VI	121.16 ± 2.59	117.16 ± 4.54	102.88 ± 5.16

MCH (Pg)	I	47.76 ± 2.01	44.52 ± 0.69	40.97 ± 0.95
	II	46.78 ± 1.22	43.21 ± 1.35	39.76 ± 0.93
	III	48.80 ± 4.04*∗*	49.00 ± 1.70*∗*	47.74 ± 2.31*∗*
	IV	47.16 ± 1.31	45.25 ± 3.72	40.13 ± 0.96
	V	49.35 ± 2.15	44.58 ± 1.40	42.24 ± 1.54
	VI	46.15 ± 1.68*∗*	43.39 ± 1.86*∗*	41.47 ± 1.76*∗*

MCHC (%)	I	38.47 ± 0.82	39.11 ± 0.50	39.04 ± 0.40
	II	38.54 ± 1.24	38.27 ± 0.47	39.44 ± 0.46
	III	37.62 ± 0.46*∗*	36.76 ± 0.46*∗*	38.16 ± 1.02*∗*
	IV	38.60 ± 0.85	38.44 ± 0.68	39.67 ± 1.00
	V	38.88 ± 1.19	37.94 ± 1.57	39.06 ± 0.62
	VI	38.09 ± 0.96	37.03 ± 0.56	40.34 ± 1.53

*∗*/*∗∗*The different superscript on the same column and parameter showed significantly different values (*P *< 0.05).

**Table 7:** Effects of 5% SAP on the haematological profile (WBC and differential leucocytes count) of broiler chickens fed a diet naturally contaminated with AF at 7, 14, and 21 days of age.

Parameter	Group	Day		
		7	14	21
WBC (× 10^3^/ *µ*L)	I	20.96 ± 6.24	22.70 ± 1.74	24.11 ± 3.92
	II	22.47 ± 3.13*∗*	24.00 ± 2.96*∗*	25.35 ± 3.72*∗*
	III	19.39 ± 48.40*∗∗*	20.70 ± 42.89*∗∗*	21.36 ± 43.66*∗∗*
	IV	22.37 ± 3.43*∗*	24.03 ± 3.55*∗*	25.40 ± 5.25*∗*
	V	22.05 ± 7.06*∗*	24.05 ± 2.42*∗*	24.98 ± 4.70*∗*
	VI	21.20 ± 4.97	22.45 ± 1.33	24.11 ± 5.84

Heterophils (× 10^3^/ *µ*L)	I	4.37 ± 2.55	5.07 ± 2.96	5.58 ± 4.91
	II	4.98 ± 3.06*∗*	5.59 ± 3.32*∗*	5.99 ± 2.15*∗*
	III	4.10 ± 4.54*∗∗*	4.51 ± 2.85*∗∗*	4.94 ± 3.09*∗∗*
	IV	4.73 ± 2.57*∗*	5.48 ± 2.99*∗*	6.09 ± 1.40*∗*
	V	4.81 ± 2.08*∗*	5.73 ± 1.18*∗*	5.58 ± 4.11*∗*
	VI	4.55 ± 3.36*∗*	5.65 ± 1.57*∗*	6.62 ± 9.17*∗*

Lymphocytes (× 10^3^/ *µ*L)	I	12.47 ± 3.22	13.73 ± 4.02	14.23 ± 4.57
	II	14.15 ± 3.50*∗*	14.96 ± 4.09*∗*	15.79 ± 3.31*∗*
	III	11.25 ± 3.96*∗∗∗*	12.34 ± 2.38*∗∗∗*	12.78 ± 4.23*∗∗∗*
	IV	13.98 ± 6.24*∗*	14.90 ± 3.07*∗*	15.87 ± 5.01*∗*
	V	12.61 ± 7.71*∗∗*	14.54 ± 1.34*∗∗*	15.24 ± 5.19*∗∗*
	VI	12.37 ± 5.54	13.66 ± 2.95	14.90 ± 2.98

Ratio H/L	I	0.35 ± 0.01	0.36 ± 0.02	0.39 ± 0.03
	II	0.35 ± 0.02	0.37 ± 0.01	0.37 ± 0.01
	III	0.36 ± 0.03	0.36 ± 0.02	0.38 ± 0.03
	IV	0.33 ± 0.02	0.36 ± 0.01	0.38 ± 0.01
	V	0.38 ± 0.02	0.39 ± 0.00	0.36 ± 0.03
	VI	0.36 ± 0.03*∗*	0.41 ± 0.01*∗*	0.44 ± 0.02*∗*

Monocytes (× 10^3^/ *µ*L)	I	2.09 ± 1.77	2.11 ± 1.15	2.41 ± 2.22
	II	1.94 ± 1.07*∗*	1.91 ± 4.80*∗*	1.56 ± 0.96*∗*
	III	1.97 ± 1.36	2.37 ± 2.30	2.17 ± 0.94
	IV	1.56 ± 3.24*∗*	1.76 ± 1.25*∗*	1.60 ± 1.35*∗*
	V	2.16 ± 2.23	2.04 ± 1.46	2.16 ± 3.98
	VI	2.08 ± 1.93	2.28 ± 2.24	2.09 ± 4.06

Eosinophils (× 10^3^/ *µ*L)	I	1.77 ± 1.52	1.55 ± 2.25	1.76 ± 4.92
	II	1.23 ± 3.92	1.43 ± 2.11	1.73 ± 2.01
	III	1.70 ± 4.40*∗*	1.03 ± 2.41*∗*	0.99 ± 3.98*∗*
	IV	1.71 ± 2.61	1.80 ± 2.78	1.61 ± 3.96
	V	2.05 ± 1.51	1.60 ± 3.01	1.74 ± 2.74
	VI	1.90 ± 3.09*∗*	0.86 ± 1.67*∗*	0.48 ± 3.47*∗*

Basophils (× 10^3^/ *µ*L)	I	0.24 ± 2.09	0.22 ± 2.04	1.12 ± 2.03
	II	0.14 ± 1.15	0.08 ± 1.25	0.25 ± 2.30
	III	3.56 ± 2.83*∗*	4.17 ± 2.65*∗*	4.63 ± 3.66*∗*
	IV	0.36 ± 5.83	0.08 ± 1.25	0.21 ± 3.02
	V	0.40 ± 2.43	0.11 ± 1.31	0.24 ± 2.21
	VI	0.28 ± 2.88	0 ± 0	0 ± 0

*∗*/*∗∗*/*∗∗∗*The different superscript on the same column and parameter showed significantly different values (*P *< 0.05).

**Table 8:** Effects of 5% SAP on the liver histopathology of broiler chickens fed a diet naturally contaminated with AF at 7, 14, and 21 days of age.

Parameter	Group	Day		
		7	14	21
Necrosis	I	0 ± 0	1.00 ± 1.09	0.50 ± 0.54
	II	0 ± 0	0.66 ± 0.51	0.50 ± 0.54
	III	0.33 ± 0.51*∗*	0.83 ± 0.98*∗*	2.16 ± 1.83*∗*
	IV	0 ± 0	1.00 ± 0.89	0.50 ± 0.54
	V	0 ± 0	0.50 ± 0.54	0.50 ± 0.54
	VI	0 ± 0	0.50 ± 0.54*∗*	1.33 ± 1.62*∗*

Perilobular inflammation	I	0 ± 0	0.33 ± 0.51	0.16 ± 0.40
	II	0 ± 0	0.16 ± 0.40	0.16 ± 0.40
	III	0 ± 0	0.83 ± 0.75*∗*	2.33 ± 1.86*∗*
	IV	0 ± 0	0.33 ± 0.51	0.16 ± 0.40
	V	0 ± 0	0.33 ± 0.51	1.16 ± 1.47
	VI	0 ± 0	0.33 ± 0.51	1.50 ± 1.37*∗*

Interlobular inflammation	I	0 ± 0	0 ± 0	0 ± 0
	II	0 ± 0	0 ± 0	0 ± 0
	III	0 ± 0	0.50 ± 0.54*∗*	1.66 ± 1.96*∗*
	IV	0 ± 0	0 ± 0	0 ± 0
	V	0 ± 0	0 ± 0	0.83 ± 1.60*∗*
	VI	0 ± 0	0 ± 0	1.16 ± 1.47*∗*

Hydrophic and/ or fatty degeneration	I	0.50 ± 1.22	0.66 ± 1.63	0.33 ± 0.51
	II	0 ± 0	0.16 ± 0.40	0.33 ± 0.51
	III	0.50 ± 0.54*∗*	1.00 ± 0.89*∗*	2.00 ± 1.09*∗*
	IV	0.16 ± 0.40	0.16 ± 0.40	0.66 ± 0.81
	V	0.16 ± 0.40	0.16 ± 0.40	1.33 ± 1.50
	VI	0.16 ± 0.40	0.16 ± 0.40	1.33 ± 1.50

Bile-duct proliferation	I	0 ± 0	0 ± 0	0.33 ± 0.81
	II	0 ± 0	0 ± 0	0.16 ± 0.40
	III	0 ± 0	0.66 ± 1.03	3.50 ± 0.83*∗∗∗*
	IV	0 ± 0	0 ± 0	0.16 ± 0.40
	V	0 ± 0	0 ± 0	0.83 ± 0.98*∗*
	VI	0 ± 0	0 ± 0	2.00 ± 1.67*∗∗*

*∗*/*∗∗*/*∗∗∗*The different superscript on the same column and parameter showed significantly different values (*P *< 0.05).

**Table 9:** Effects of 5% SAP on the kidney histopathology of broiler chickens fed a diet naturally contaminated with AF at 7, 14, and 21 days of age.

Parameter	Group	Day		
		7	14	21
Necrosis	I	0 ± 0	0.16 ± 0.40	0.50 ± 0.54
	II	0 ± 0	0.16 ± 0.40	0.33 ± 0.51
	III	0 ± 0	1.00 ± 0.89*∗*	2.16 ± 0.75*∗*
	IV	0 ± 0	0.16 ± 0.40	0.50 ± 0.54
	IV	0 ± 0	0.16 ± 0.40	0.66 ± 0.81
	VI	0 ± 0	0.16 ± 0.40	0.66 ± 0.81

Degeneration	I	0 ± 0	0.33 ± 0.51	0.50 ± 0.54
	II	0 ± 0	0.16 ± 0.40	0.33 ± 0.51
	III	0 ± 0	0.83 ± 0.75*∗*	1.50 ± 1.04*∗*
	IV	0 ± 0	0.50 ± 0.83	0.66 ± 1.21
	V	0 ± 0	0.33 ± 0.51	0.50 ± 0.83
	VI	0 ± 0	0.33 ± 0.51	0.50 ± 0.83

Inflammation	I	0 ± 0	0 ± 0	0 ± 0
	II	0 ± 0	0 ± 0	0 ± 0
	III	0.16 ± 0.40*∗∗*	1.00 ± 0.89*∗∗*	1.33 ± 1.21*∗∗*
	IV	0 ± 0	0 ± 0	0.16 ± 0.40
	V	0 ± 0	0.50 ± 0.83*∗*	0.50 ± 0.83*∗*
	VI	0 ± 0	0.50 ± 0.54*∗*	0.50 ± 0.54*∗*

*∗*/*∗∗*The different superscript on the same column and parameter showed significantly different values (*P *< 0.05).

## Data Availability

The data used to support the findings of this study are available from the corresponding author upon request.
